# DNA hypomethylation mediates immune response in pan-cancer

**DOI:** 10.1080/15592294.2023.2192894

**Published:** 2023-03-22

**Authors:** Chunlong Zhang, Qi Sheng, Ning Zhao, Shan Huang, Yuming Zhao

**Affiliations:** a College of Information and Computer Engineering, Northeast Forestry University, Harbin, Heilongjiang, China; b College of Bioinformatics Science and Technology, Harbin Medical University, Harbin, Heilongjiang, China; c The Second Affiliated Hospital, Harbin Medical University, Harbin, Heilongjiang, China

**Keywords:** DNA methylation, pan-cancer, immune, hypomethylation, biomarker

## Abstract

Abnormal DNA methylation is a fundamental characterization of epigenetics in cancer. Here we demonstrate that aberrant DNA methylating can modulate the tumour immune microenvironment in 16 cancer types. Differential DNA methylation in promoter region can regulate the transcriptomic pattern of immune-related genes and DNA hypomethylation mainly participated in the processes of immunity, carcinogenesis and immune infiltration. Moreover, many cancer types shared immune-related functions, like activation of innate immune response, interferon gamma response and NOD-like receptor signalling pathway. DNA methylation can further help identify molecular subtypes of kidney renal clear cell carcinoma. These subtypes are characterized by DNA methylation pattern, major histocompatibility complex, cytolytic activity and cytotoxic t lymphocyte and tumour mutation burden, and subtype with hypomethylation pattern shows unstable immune status. Then, we investigate the DNA methylation pattern of exhaustion-related marker genes and further demonstrate the role of hypomethylation in tumour immune microenvironment. In summary, our findings support the use of hypomethylation as a biomarker to understand the mechanism of tumour immune environment.

## Introduction

The human immune system can eliminate cancer cells through acquired immune response implemented by immune cells. Nevertheless, clinically detectable tumour is often caused by a failure of immunosurveillance. For example, *FOXP3* is an important intracellular molecule for regulatory T cell’s (Treg) development and function, which is regarded as a key marker for Treg [1]. The *FOXP3*+ Treg suppresses antitumor response and leads to immunological tolerance for host tissues [2]. Identifying these immune escape mechanisms can provide opportunities for cancer immunotherapy, lifting immune suppression and restoring antitumor immunity. *FOXP3*+ Treg is thought as a potential therapeutic and prognostic factor, which has been reported in several cancers such as breast, ovarian, hepatocellular, lung and cervical cancers [3–7]. Despite the effective immunotherapy for some patients, many patients do not benefit from immune treatment, which may be caused by insufficient immunogenicity, tumour cell-intrinsic and extrinsic mechanism and acquired resistance [8,9].

Epigenetics can change the function of the genome without altering the sequence of nucleotides [10]. DNA methylation is one of the important mechanisms in epigenetics. Cytosine residues are methylated by adding methyl groups to the fifth carbon atom [11]. Gene transcription is the major area for cancer research, treatment and drug discovery [12]. Gene transcription is regulated through DNA methylation, which generally induces gene repression when methylated at CpG dinucleotides of promoter region [13,14]. There is evidence that DNA methylation plays a key role in tumorigenesis based on genome-wide patterns of DNA methylation [15,16]. With the development of assessing genome-wide CpG methylation profiles, epigenomic studies of genetic diseases such as cancer have become possible [17–19]. Currently, these data are mostly used to evaluate CpG sites with consistent methylation abnormity in cancer for differential methylation studies. Methylation alterations have been found to be a hallmark in tumour development [15,20]. Besides, the function of immune cells has been studied in terms of DNA methylation. In general, the promoter region of cytokine loci (*IFNG* and *IL2*) is demethylated in activated T cell, which demonstrates the potential role of DNA methylation in immune microenvironment [21–23]. DNMT1 is the DNA methyltransferase enzyme in mammals, which can influence the level of DNA methylation [24]. DNMT1-deficient haematopoietic stem cells are biased towards myeloerythroid lineages, suggesting that DNA methylation is critical to lymphoid development [25]. DNA methylation in tumour tissue from cutaneous metastases predicts the therapy response to immune checkpoint inhibition for stage IV metastatic melanoma [26]. Multiple cancers are found to have methylation of the *PDL1* promoter that regulates the expression of *PDL1* negatively and is associated with patients’ prognosis [27–29]. According to the relationship between DNA methylation and immune system in cancer, epigenetic therapy has been developed and proved to treat haematological and solid tumours successfully, and there is an ongoing clinical trial to develop drugs and therapy combinations [30,31]. Moreover, treatment with DNMTi improves the sensibility of tumour cells to chemotherapy, transplantation or immunotherapy [32]. Therefore, understanding how DNA methylation affects the immune microenvironment can help to explore cancer mechanisms and improve cancer immunotherapy treatment.

Even though many studies have been conducted about DNA methylation and immune microenvironment, a systematic investigation concerning DNA methylation and immune microenvironment remains absent. In this study, we aimed to analyse the genome-wide DNA methylation profiles of pan-cancer to identify the epigenetic mechanisms of immune microenvironment. Then, we used various bioinformatic methods to explore the function of immune-related genes. Finally, we further analysed the methylated pattern of exhaustion-related marker genes. We put forward our study to identify marker genes for immune microenvironment, and provided theoretical foundation for other researches focusing on diagnosis, prognosis, and treatment of cancer.

## Materials and methods

### Data availability

In order to further verify methylated pattern of immune cell, we downloaded the Illumina Infinium Human Methylation450 (HM450K bead array) BeadChip of 32 tumours through the TCGA database (https://portal.gdc.cancer.gov/) and GEO database (https://www.ncbi.nlm.nih.gov/geo/). HM450K BeadChip data covered approximately 480,000 CpG sites. The annotation information of CpG sites and coding genes were extracted from the annotation file of HM450K, which came from the hg19 reference genome. To confirm the specific DNA methylation level of cancer patients, it was necessary to choose the cancer types which had normal samples for DNA methylation. We selected the cancer types whose number of normal samples were no less than four for subsequent analysis. Finally, 16 cancers (BLCA, BRCA, CHOL, COAD, ESCA, HNSC, KIRC, KIRP, LIHC, LUAD, LUSC, PAAD, PRAD, READ, THCA, UCEC) were obtained (Table 1).

**Table 1.** The cancer information.

Abbreviation	Cancer Name	Tumor (Methylation)	Normal (Methylation)	Tumor (Expression)
BLCA	Bladder Urothelial Carcinoma	407	21	408
BRCA	Breast Invasive Carcinoma	781	96	1091
CHOL	Cholangiocarcinoma	36	9	36
COAD	Colon Adenocarcinoma	293	38	456
ESCA	Esophageal Carcinoma	184	16	161
HNSC	Head and Neck Squamous Cell Carcinoma	526	50	500
KIRC	Kidney Renal Clear Cell Carcinoma	318	160	530
KIRP	Kidney Renal Papillary Cell Carcinoma	272	45	288
LIHC	Liver Hepatocellular Carcinoma	375	50	371
LUAD	Lung Adenocarcinoma	438	32	513
LUSC	Lung Squamous Cell Carcinoma	355	42	501
PAAD	Pancreatic Adenocarcinoma	184	10	177
PRAD	Prostate Adenocarcinoma	498	50	495
READ	Rectum Adenocarcinoma	97	7	166
THCA	Thyroid Carcinoma	429	46	502
UCEC	Uterine Corpus Endometrial Carcinoma	407	21	543
GSE113501 (KIRC)	Kidney Renal Clear Cell Carcinoma	132	22	-

### Quantification of DNA methylation and gene expression

The HM450K BeadChip data contained not only CpG sites located in euchromosome, but also CpG sites located in sex chromosome and near single nucleotide polymorphism (SNP) sites. The instability of CpG sites near SNPs and sex chromosome could interfere with our analysis, so both types of sites were removed before analysis. Next, we used the beta value to define the methylation level of each CpG sites as follows:

$$\beta_{i}=\frac{Methy_{i}}{\left(Methy_{i}+UnMethy_{i}\right)}$$

Where $\beta_{i}$, $Methy_{i}$ and $UnMethy_{i}$ represented the methylation level, the intensity of methylation and the intensity of unmethylation of CpG_i_, respectively [33].

Based on the global observation of CpG sites, we found that some CpG sites had missing values in cancer samples. Therefore, we chose the CpG sites whose missing values were less than 40% in each cancer sample, and the missing CpG sites were filled with the mean of each CpG site. Finally, the total number of CpG sites covered by all cancers was about 390,000. According to the transcriptional information of HM450K annotation, CpG sites were located in different transcription-related regions, such as 1stExon, 3’UTR, 5’UTR, Gene Body, TSS1500 and TSS200. Previous studies have confirmed that when DNA methylation occurred at the CpG site in promoter region, DNA methylation would inhibit the binding of transcription factors and decrease mRNA expression in downstream. Therefore, DNA methylation in the promoter region was of greater interest. We defined 1stExon, 5‘UTR, TSS1500 and TSS200 regions as promoter regions, and extracted the CpG sites in the corresponding regions for subsequent analysis.

The promoter region of a gene usually covered multiple CpG sites, and the expression of downstream coding genes was also affected by these sites. In order to explore the regulation mechanism of multiple CpG sites on downstream coding genes, the RNA-seq expression profiles of 16 cancers were downloaded from TCGA database. The mRNA expression profile covered about 19,000 genes, and RPKM (Reads Per Kilobase of exon per Million reads mapped) was used to quantify the expression level of each mRNA. The expression level of mRNA was normalized based on the log2(RPKM+1) conversion [34]. The mRNA expression profiles of TCGA database were identified by the ENSG id. In order to match the previous analysis and remove the confusing information, the human gene annotation file was downloaded from the GENCODE database (https://www.gencodegenes.org/) to obtain the corresponding relationship between ENSG id and the gene symbol. The entrez id, gene symbol and ENSG id were standardized for subsequent analysis.

### Differential analysis of DNA methylation

In order to verify the DNA methylation patterns of cancer, we used the student’s T test method to make differential analysis. The threshold value of differentially methylated sites was *p*< = 0.05 and $|Ave_{Cancer}-Ave_{Normal}|\geq 0.1$. The CpG sites with $Ave_{Cancer}-Ave_{Normal}\geq 0.1$ and $Ave_{Cancer}-Ave_{Normal}\leq -0.1$ were regarded as hypermethylated and hypomethylated sites, respectively [35].

### The relationship between methylation and expression

To assess the relationship between DNA methylation and gene expression, we used the multiple linear regression to evaluate the association between DNA methylation and gene expression:

y=Xβ+ε

Where, y was the vector $\left[y_{1},\;y_{2},\ldots ,y_{n}\right]$, and $y_{n}$ represented the expression level of genes in sample n; X was the matrix $\left[X_{1},\;X_{2},\ldots ,X_{p}\right]$, and $X_{p}$ represented the methylation value of CpG site p; X_p_ was the vector $\left[x_{1p},\;x_{2p},\ldots ,x_{np}\right]$, and $x_{np}$ represented the methylation value of site p in sample n; β was the vector $\left[\beta_{1},\;\beta_{2},\ldots ,\beta_{p}\right]$, and $\beta_{p}$ represented the regression coefficient of site p; ε was a constant term [36,37].

After performing multiple linear regression, the threshold of multiple linear regression was defined as 0.05. After regression analysis, 10568 genes with significant regulatory relationships were covered.

### The functional enrichment analysis of methylation-related genes

To reveal the mechanism of methylation-related genes, we used the GSEA software to evaluate the functions of the significant genes through multiple linear regression [38]. Functional enrichment analysis was carried out using the pre-rank function of the GSEA software. Since the pre-rank function needed to sort the genes before analysis, we integrated the *p* value of multiple linear regression and methylated difference value ($delta=Ave_{Tumor}-Ave_{Normal}$) in promoter region to sort the genes in 16 cancers. The formula was as follows:

$$RS_{i}=-\log\left(p_{i}\right)*sign\left(delta_{i}\right)$$

Where $p_{i}$ was the *p* value of gene i, and $delta_{i}$was the difference value of gene i between tumour and normal samples [39–41]. The higher the absolute value of RS was, the significant relationship between methylation and expression was. And the genes with high RS score were worth further investigation.

### Immunological score

Major Histocompatibility Complex (MHC), Cytolytic Activity (CYT) and Cytotoxic T Lymphocyte (CTL) were used to predict immune response. The HMC was calculated as:

$$MHC_{m}=\left(\sum exp_{i}\right)/9$$

Where, $exp_{i}$ was the expression level of gene i in sample m, and i was one of nine genes (*HLA-A*, *PSMB9*, *HLA-B*, *PSMB8*, *HLA-C*, *B2M*, *TAP2*, *NLRC5*, and *TAP1*). These nine genes could represent the core gene set of MHC-I [42].

The CYT was calculated as:

$$CYT_{m}=\left(\sum exp_{i}\right)/2$$

Where, $exp_{i}$ was the expression level of gene i in sample m, and i was one of two genes (*GZMA* and *PRF1*). These two genes were key factors of cytolysis and highly expressed in CD8+ T cells [43].

The CTL was calculated as:

$$CTL_{m}=\left(\sum exp_{i}\right)/3$$

Where, $exp_{i}$ was the expression level of gene i in sample m, and i was one of three genes (*GZMA*, *GZMB* and *PRF1*). These three genes can estimate T cell toxicity and immune effect [44].

### Survival analysis

Survival analysis was carried out through the webserver Survivalmeth, in which the Maxstat model was used to estimate the optimal cut-off threshold to classify the subtypes [45]. The Kaplan-Meier (K-M) curve was used to predict the prognosis between different subtypes.

## Result

### The common and specific modes of abnormally epigenetic regulation among cancers

**Figure 1.**
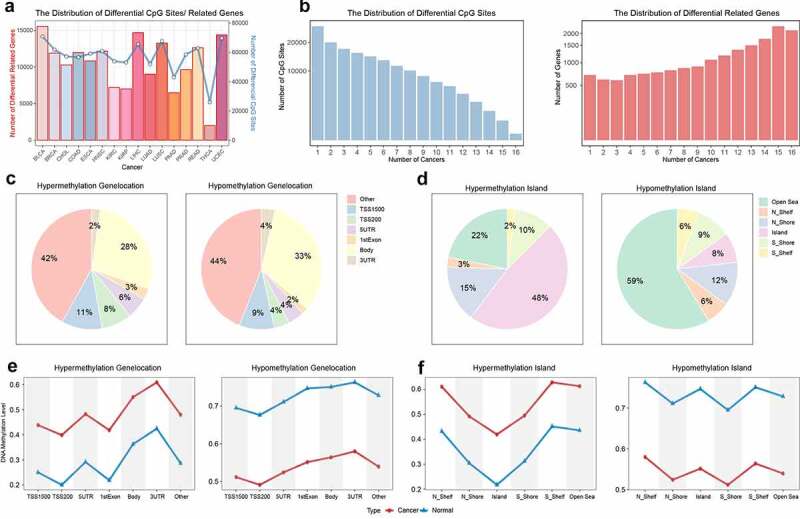
The distribution of differential methylation. a. The distribution of differential CpG sites and genes. The line plot represented the number of differential CpG sites. The bar plot represented the number of genes whose promoter regions had differential CpG sites. b. The coverage of CpG sites and genes in 16 cancers. The y axis represented the number of differential CpG sites and related genes. The x axis represented the number of cancers sharing the differential CpG sites and related genes. c. The distribution of differential CpG sites in transcriptional regions. d. The distribution of differential CpG sites in island regions. e. The methylation level of differential CpG sites in different transcriptional regions. f. The methylation level of differential CpG sites in different island regions. The red and blue represented tumor and normal samples respectively.

In order to characterize the heterogeneous performance of DNA methylation, we analysed the sample information among different cancers (SupplementaryFigure 1). The distribution of tumour malignant level and age were different among different cancers, which could display the tumour heterogeneity and provide the foundation for further methylation analysis among pan-cancer. Then we made differential analysis between tumour and normal samples. 56506 differentially methylated CpG sites were identified among 16 cancers (Figure 1). Through the distribution of differentially methylated sites, we found that abnormal hypermethylation were more frequent in the promoter region than hypomethylation (hypermethylation: 28%, hypomethylation 19%), and previous studies have confirmed the common phenomenon that the promoter region was abnormally hypermethylated and the whole genome was abnormally hypomethylated in cancers. Based on the CpG island information, about 78% of abnormally hypermethylated sites occurred in the CpG island area and its adjacent areas (shelf and shore), and the proportion of the CpG island area was even as high as 48%. Most of the abnormal hypomethylation occurred in the open sea area (59%). The high enrichment of abnormal hypermethylation in the promoter CpG island region has been demonstrated by previous studies [46].

Next, we also visualized the DNA methylation pattern of differentially methylated sites. The result showed that abnormal hypermethylation mainly occurred at the original hypomethylated sites (methylation level < = 0.5), and abnormal hypomethylation mainly occurred at the original hypermethylated sites (methylation level> = 0.7). However, the hypomethylated sites rarely became much lower, and the hypermethylated sites rarely became much higher. The result demonstrated the phenomenon of global hypomethylation, and indicated the negative regulation mechanism of abnormal DNA methylation that the hypomethylation of oncogenes and the hypermethylation of tumour suppressor genes often influenced occurrence and development of cancer.

### Abnormal DNA methylation regulates cancer immune-related functions

**Figure 2.**
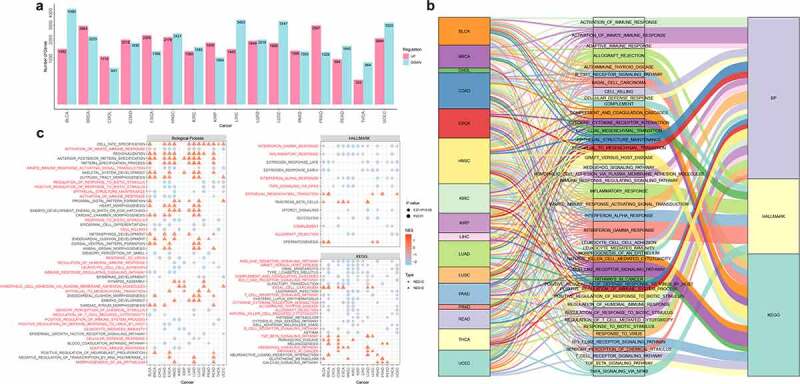
**The analysis of methylation-related genes**. a. The number of methylation-related genes through the multiple linear regression analysis. The UP and DOWN meant hypermethylation and hypomethylation compared with normal samples. b. The distribution of cancer-related functions in different cancers. c. The coverage of functions in different cancers. The red name meant strong association with cancer development. The circle meant the NES score was less than zero and the function was regulated by hypomethylation. The triangle meant the NES score was greater than zero and the function was regulated by hypermethylation. The color represented the scale of NES score. The node size represented the significance of *p* value.

To understand the regulation mechanism of DNA methylation in cancer, the corresponding mRNA expression profiles were downloaded from TCGA. Then we used linear regression analysis to explore the relationship between DNA methylation and expression. 10568 correlated genes were collected in 16 cancers (Figure 2a). Next, we used RS score to make functional enrichment analysis through GSEA. The KEGG pathway, biological process (BP) and hallmark were utilized to evaluate the function of significant genes (Figure 2b). Subsequently, the normalized enrichment score (NES) and adjusted *p* value were used to evaluate the GSEA functional enrichment results (adjusted *p* value < = 0.05). When the NES value was greater than zero or less than zero, it meant that hypermethylation or hypomethylation has affected the function. 16 cancers were mainly annotated to 382 biological processes (NES>0: 176, NES<0: 206), 135 KEGG pathways (NES>0: 56, NES<0: 79) and 54 hallmarks (NES>0: 20, NES<0: 34). We found the functions regulated by hypomethylation were more correlated with immunity and cancer. Then we collected functions located in at least three cancers to explore the relationship between them. The NES score showed that different types of cancer had consistent methylation patterns and high coverage of many functions, which included 47 biological processes, 13 hallmarks and 28 KEGG pathways. Many of these functions were closely related to cancer and immunity, such as the activation of innate immune response, biological stimulus response, epithelial structure maintenance, cell killing, response to virus, humoral immunity, leukocyte-mediated immunity, cellular defence response, interferon response, inflammatory response, TNF signal, NOD receptor Body signalling, T cell receptor signalling, cytokine interaction, natural killer cytotoxicity, and B cell signalling pathways. These results showed that abnormal methylation might played an important role in cancer and immunity.

In order to investigate the impact of abnormal hypermethylation/hypomethylation on cancer development, the number of functions regulated by hypermethylation/hypomethylation was sorted out (Figure 2c). 19 out of 22 biological processes were regulated by hypomethylation, while only three were regulated by hypermethylation. six out of seven hallmarks were regulated by hypomethylation; and 10 out of 14 pathways were also regulated by hypomethylation. Next, we analysed the top functions in the three functional categories. In biological process, the second-ranked activation of innate immune response (covering 10 cancers) was closely related to the immune response. The first step in fighting cancer cells was activating innate immune cells, and they could also work together with adaptive immune cells [46,47]. In hallmark, the first-ranked interferon gamma response (covering 11 cancers) and the second-ranked inflammatory response (covering 9 cancers) were closely related to the immune response. Interferon gamma coordinated the innate and adaptive immune response to viruses, bacteria and cancer. Inflammation could activate the immune system, while chronic inflammation could deplete the immune response [48,49]. Among the KEGG pathways, the first-ranked NOD-like receptor signalling pathway (covering eight cancers) and the second-ranked graft-versus-host disease (covering nine cancers) were closely related to the immune response. NOD-like receptors have been confirmed as an important regulator of inflammation-related tumorigenesis, angiogenesis, cancer cell chemotherapy and drug resistance [50]. Moreover, these five immune-related functions were all regulated by abnormal hypomethylation, proving that abnormal hypomethylation could lead to the imbalance of immune response and promote the development of cancer [51,52].

**Figure 3.**
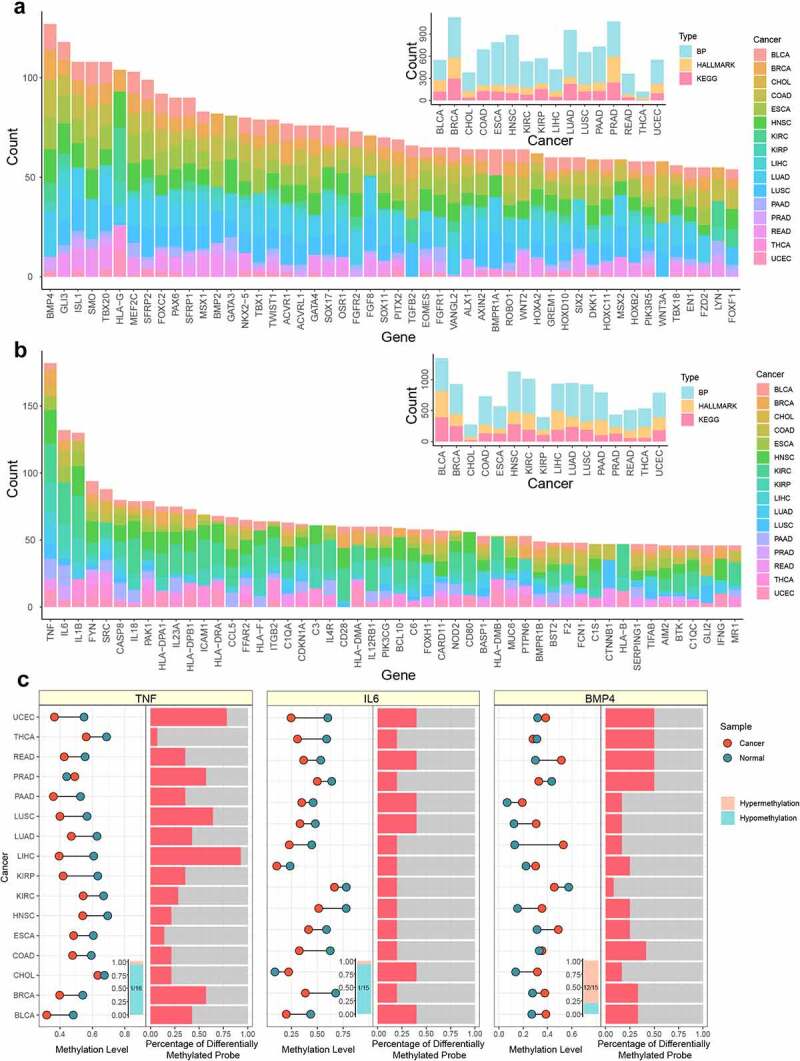
The analysis of top genes in functional enrichment. a. The distribution of genes in functional enrichment of different cancers with NES>0. The large bar plot represented the number of genes in functional enrichment with NES>0, and the small bar plot represented the number of functions in different cancers. b. The distribution of genes in functional enrichment of different cancers with NES<0. The large bar plot represented the number of genes in functional enrichment with NES<0, and the small bar plot represented the number of functions in different cancers. c. The methylation level of top genes in functional enrichment. The dot plot represented the average methylation level of tumor and normal samples in different cancers. The red node and green node represented tumor and normal sample, respectively. The bar plot in the bottom represented the number of cancers where the gene was differential. The orange and blue represented hypermethylation and hypomethylation, respectively. The bar plot in the right side represented the percentage of differentially methylated CpG sites in promoter region.

Next, the gene frequencies of functions with NES greater than zero and less than zero were integrated (Figure 3a and b). For functions with NES less than zero, *TNF* (Tumour Tecrosis Factor) was the most frequent. *TNF* was a superfamily of cytokines, and its family members were involved in the maintenance and homoeostasis of the immune system, inflammation and host defence. *TNF* was also detected in cancer and might lead to a poor prognosis of cancer [53]. Moreover, many studies have confirmed that the second-ranked gene interleukin *IL6* was also an important multi-functional cytokine involved in tumour growth and metastasis [54,55]. The high expression of both genes was detected in many cancers, and they were often used as the marker for cancer diagnosis and poor prognosis [56]. For functions with NES greater than zero, *BMP4* (Bone Morphogenetic Protein 4) was the most frequent. Hypermethylation of this gene has been associated with poor prognosis for cancer patients in many studies [57,58]. Afterwards, the average methylation level of these three genes in promoter region were visualized between tumour and normal samples. *TNF* was significantly hypomethylated in 15 cancers, *IL6* was significantly hypomethylated in 14 cancers, and *BMP4* was significantly hypermethylated in 12 cancers, indicating that these important genes were regarded in a stably consistent manner among different types of cancer. We also counted the number of abnormally methylated CpG sites in promoter region, showing that despite the average methylation level decreasing consistently in promoter region, there was still a specific distribution of differential CpG sites among cancers (Figure 3c).

### DNA hypomethylation genes regulate immune infiltration

**Figure 4.**
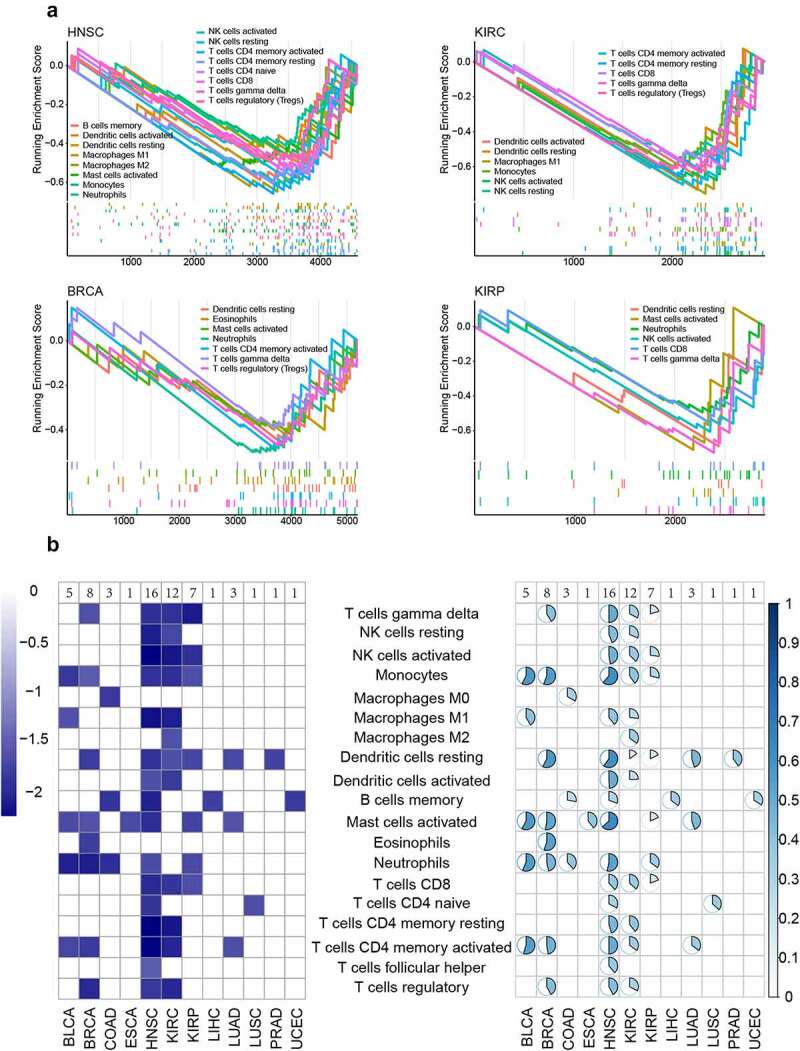
The immune infiltration regulated by hypomethylation. a. The GSEA result of immune infiltration. The y axis represented the NES score of GSEA result. The x axis represented the rank of infiltration-related marker genes. b. The NES result of immune infiltration. The left part represented the NES score of each significant immune infiltration result in different cancers. The right part represented the percentage of differentially methylated marker genes for each significant immune infiltration result in different cancers.

According to these results, abnormal methylation, especially hypomethylation, played an important role in cancer and immune response. To examine the impact of abnormal DNA methylation on immunity, immune infiltration was investigated. The marker genes of 22 immune cells were downloaded from the CIBERSORT database, and used the RS score mentioned above to perform GSEA enrichment analysis on marker gene set for immune cells (*p* <  = 0.05) [59]. 19 immune cells were significantly enriched in at least 1 type of cancer, and many marker genes were differentially methylated in each type of immune cell. HNSC (16), KIRC (12), BRCA (8), KIRP (7) and BLCA (5) were enriched in more than five kinds of immune cells (Figure 4a). Further, we found that the enrichment results mainly had a negative NES score (Figure 4b). This result indicated that immune infiltration was mostly affected by differential hypomethylation, and hypomethylation in the promoter region caused immune instability.

**Figure 5.**
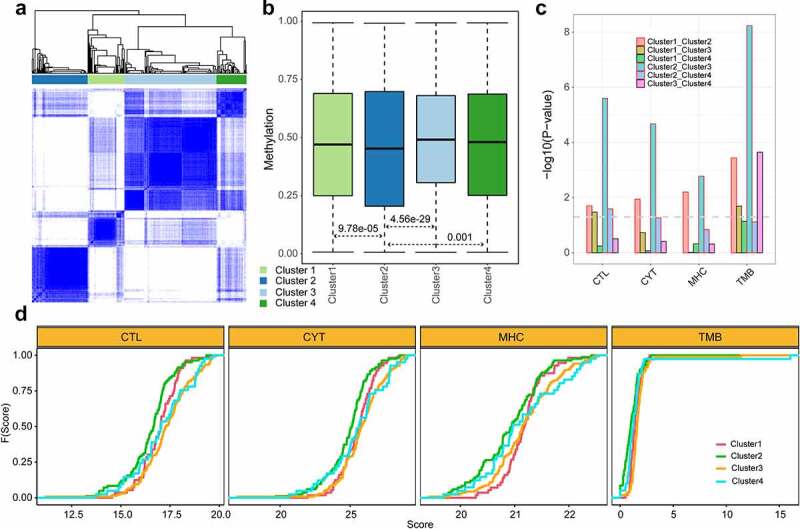
**The characterization of KIRC subtypes**. a. The heatmap of the matrix of co-occurrence proportions for KIRC. b. The methylation levels of different subtypes. c. The *p* value of two-sides Wilcoxon’s rank-sum test for TMB and immune scores between each two subtypes. The line represented *p*=0.05. d. The cumulative distribution of TMB and immune scores for patients of different subtypes. The y axis represented the cumulative percentage of score. The x axis represented the immune score and TMB.

KIRC is an immune-related cancer, so we compared the immune response of different subtypes of KIRC using the methylation profiles. The subtypes were defined based on the package ‘ConsensusClusterPlus.’ The samples were classified into four subtypes (Figure 5a). To assess the immune effects among four subtypes, each subtype was evaluated through multiple immune scores: MHC, CYT, CTL and tumour mutation burden (TMB). MHC, CYT and CTL were the key biomarkers for predicting immune response, and TMB was regarded as a potential biomarker for immunotherapy [60,61]. The cluster2 showed significantly lower methylation pattern, immune score and TMB than other three clusters (P <  = 0.05, two-sides Wilcoxon’s rank-sum test) (Figure 5b, c and d). The results showed that patients with low methylation levels had a much worse immune status and were less likely to respond to immunotherapy.

### DNA methylation regulates the expression of exhaustion-related genes

**Figure 6.**
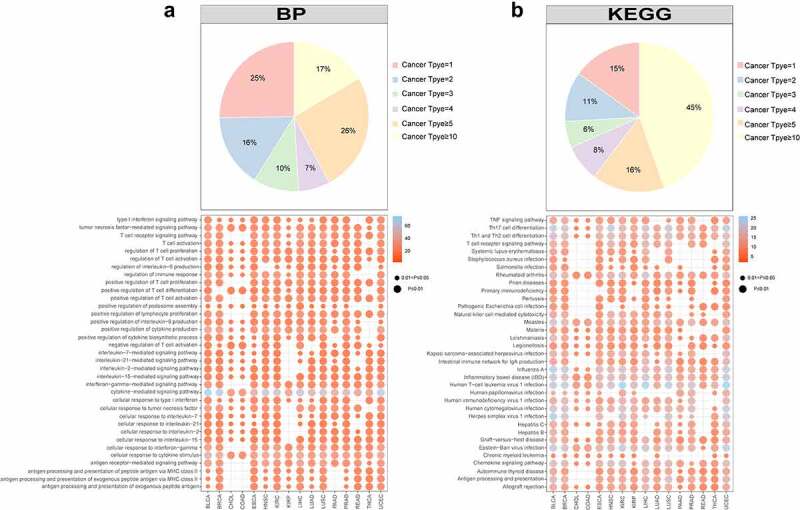
**The functional enrichment analysis of exhausted marker genes**. a. The result of biological process (BP). b. The result of KEGG pathway. The pie plot represented how many cancer types each function covered. The dot plot represented the detailed coverage information between cancer types and functions. The node size represented the significance of *p* value. The node color represented the number of marker genes enriched in the function.

Several studies have demonstrated that aberrant DNA methylation can cause abnormal immune response and be associated with inflammation [62–64]. There is evidence that inflammation is one of the main causes of T cell exhaustion, so DNA methylation may also contribute to the exhaustion process [65]. In order to investigate the impact of abnormal DNA methylation on immune exhaustion, we used the keywords ((‘t-lymphocytes’ OR ‘T cell’) AND (‘exhaust’ OR ‘exhausted’ OR ‘exhaustion’)) to search exhaustion-related publications from PubMed. Then, we carefully checked the abstracts or the full texts manually to obtain 1,285 candidate genes (SupplementaryTable 1). Then we used differential methylation analysis to analyse 1,285 genes. It was found that 753 genes were differentially methylated in at least 1 cancer and 559 genes in at least 5 cancers, suggesting that multiple cancers shared the same markers of exhausted T cells. In total, 702 genes were closely associated with gene expression in at least 1 cancer, while 328 genes were closely associated with gene expression in at least 5 cancers (SupplementaryFigure 2). The differential genes included 16 inhibitory receptors (*CTLA4*, *PDCD1*, *CD38* and *TIGIT*), 61 transcription factors (*EOMES*, *TOX* and *BATF*) and 43 cytokines (*TNF*, *IL6* and *IFNG*). The functional enrichment analysis was then performed on 328 genes using enrichr [66]. We extracted biological processes and the KEGG pathways, many of which were closely related to immunity (Figure 6). There was a high prevalence of immune processes in cancer, as 17% of biological processes occurred in more than 10 cancers, and 45% of KEGG pathways occurred in more than 10 cancers, suggesting that the coding genes affected by DNA methylation were stable in different cancers.

### Exhausted hub genes are more likely to be regulated by DNA methylation

**Figure 7.**
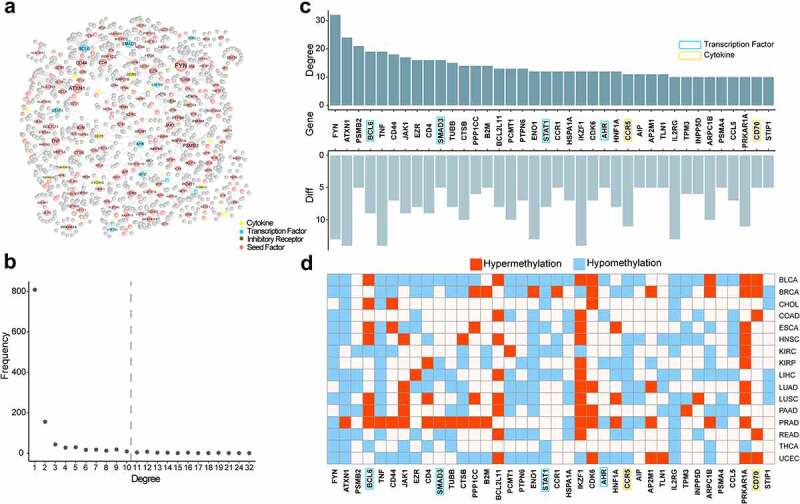
The PPI network of exhausted marker genes. a. The PPI network of exhausted marker genes. Yellow, blue, green and pink represented cytokine, transcription factor, inhibitory receptor and common seed factor, respectively. Grey represented the genes interacted with seed genes. b. The distribution of node degree. c. The characteristics of hub genes in PPI network. The top bar plot represented the degree distribution of hub genes, the bottom bar plot represented the count of cancers covered by differentially methylated genes. d. The regulation tendency of differentially methylated genes in different cancers.

To explore the interactions of these maladjusted genes and their role in cancer, we used these maladjusted genes to construct a protein-protein interaction (PPI) network associated with T cell exhaustion. To obtain a comprehensive and reliable PPI network, we downloaded experimentally validated PPI pairs from BioGRID, HPRD, Bioplex, and Huri databases. We input 702 differential genes into the background network as seed nodes and extracted pairs interacted with seed nodes to construct the PPI network of T cell exhaustion (Figure 7a). There were four classes of exhaustion-related genes in PPI network: 1. Transcription factors; 2. Cytokines; 3. Inhibitory receptors; 4. Common exhaustion-related genes. A total of 1,350 gene nodes and 1,385 relationship pairs were obtained.

In order to determine the biological significance of PPI network, we analysed the degree of all nodes in PPI network. A node’s degree represented how many nodes it was connected to. Based on the degree distribution of the nodes from 1 to 32, 38 hub genes had a degree no less than 10 (Figure 7b). We found that these hub genes were regulated by DNA methylation in at least 5 cancers, and 11 hub genes were in at least 10 cancers (Figure 7c). The genes included 32 common exhaustion-related genes, 4 transcription factors, and 2 cytokines. The highest level, *FYN* (Fyn Proto-Oncogene), was regulated by DNA methylation in 13 cancers. *FYN* was a member of the tyrosine kinase gene family, which has been linked to hepatitis and autoimmune diseases. Its expression was found to be high in gliomas, where high level could enhance immunosuppression and promote tumour growth. Besides, *FYN* was also highly expressed in T cell exhaustion and was thought to be a biomarker in T cell exhaustion [67]. According to this study, DNA methylation level of *FYN* in the promoter region decreased in 13 cancers and significantly regulated transcription downstream of the gene. *BCL6* (B cell lymphoma 6) was an oncogene in B cell lymphoma. It blocked the terminal differentiation of B cell through inhibiting proliferation and DNA damage checkpoints and led to the malignant phenotype [68]. Recent study also found that *BCL6* has been implicated in the development of type B acute lymphocyte leukaemia, chronic myeloid leukaemia, breast cancer and non-small-cell lung carcinoma, suggesting that it might be a potential drug target. *BCL6* has also been found to be significantly overexpressed in T cell exhaustion and closely associated with exhausted phenotypes in recent studies [69–71]. The *CCR5* (C-C chemokine receptor type 5) has been shown to play an important role in human disease, and mediate the physiological functions of immune cells such as T cell, macrophage, eosinophil granulocyte, myeloid suppressor cell, microglia and dendritic cell. As a result of *CCR5* overexpression, calcium signalling was activated, which enhanced regulatory T cell differentiation and facilitated their migration to inflammatory sites, and led to the T cell exhaustion [72,73]. Besides, hub genes showed hypomethylation pattern in most cancers (Figure 7d). These results showed that exhaustion-related marker genes played an important role in cancer and hypomethylation in promoter regions caused instability in their expression patterns, which disrupted the immune microenvironment in cancer and were regarded as potential targets for cancer treatment.

**Figure 8.**
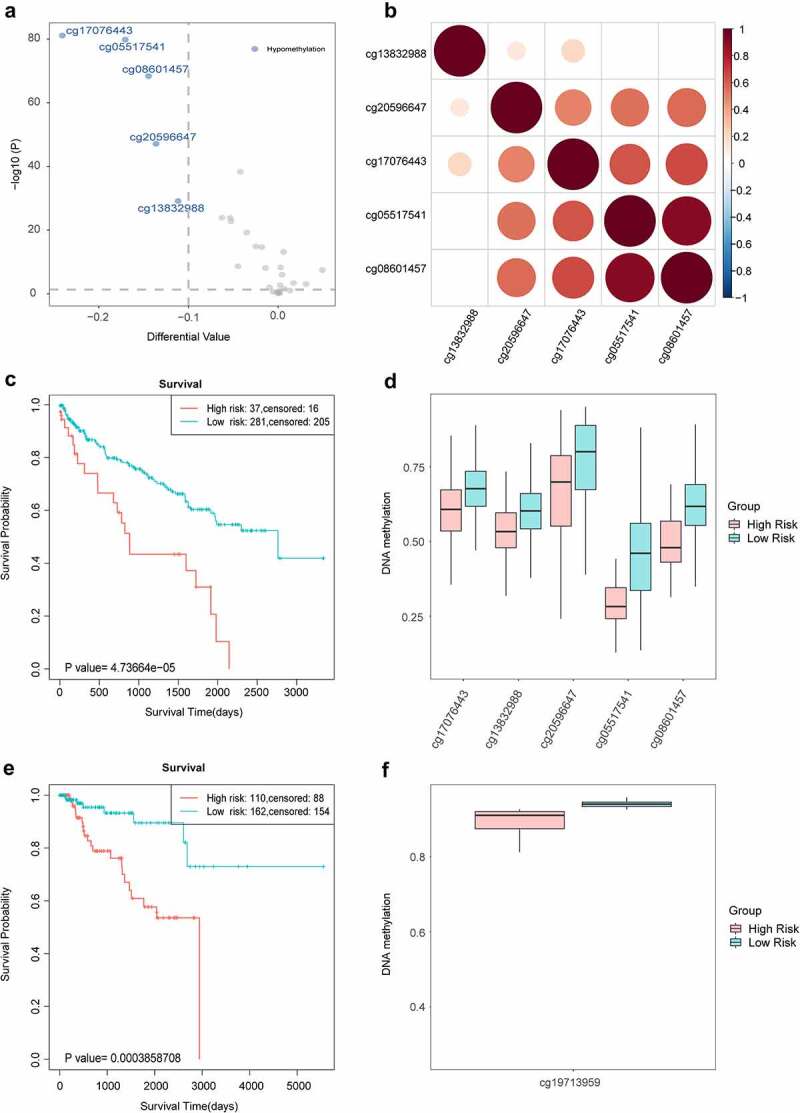
The survival analysis of kidney cancer. a. The volcano plot of differentially methylated sites in KIRC. The blue represented the significant CpG sites. b. The correlation among significantly differential sites. c. The survival curve of KIRC. The red and blue represented the high-risk group and low-risk group, respectively. d. The comparation of methylation levels between high-risk group and low-risk group for KIRC. E. The survival curve of KIRP. F. The comparation of methylation levels between high-risk group and low-risk group for KIRP.

KIRC has been demonstrated to be associated with immune microenvironment, then we decided to analyse the role of T cell exhaustion in KIRC. Firstly, we used 30 CpG sites in promoter region of *FYN* to make differential analysis (*P* <  = 0.05 and |different value| = 0.1, student’s T test) and 5 CpG sites were differential between tumour and normal samples. These five CpG sites were significant hypomethylation and showed strong relationship among each other (Figure 8a and b). Secondly, we used these five CpG sites to make survival analysis through SurvivalMeth. We calculated regression coefficients of CpG sites by COX regression analysis: Risk Score = (−1.68)*cg05517541 + (1.94)*cg08601457 + (−0.93)*cg13832988 + (−1.52)*cg17076443 + (−0.09)*cg20596647. We found coefficients of four CpG sites were less than 0, which demonstrated the risk factor of hypomethylation for cancer prognosis. The prognosis of cancer patients was evaluated by K-M curve and logrank test after grouping by Maxstat. These five CpG sites could distinguish high-risk group from low-risk group significantly (*P* = 4.737e-05) (Figure 8c). We then compared the changes of methylation levels between high-risk and low-risk group. The methylation levels of five CpG sites in high-risk group were significantly lower than those in low-risk group (student’s T test), which further revealed the influence of abnormal hypomethylation on the poor prognosis of cancer patients (Figure 8d). In order to demonstrate the role of exhausted hub genes on prognosis, we made survival analysis with each hub gene in pan-cancer, respectively (SupplementaryFigure 3). The result showed that most of these genes were associated with prognosis significantly in pan-cancer, and *FYN* was associated with prognosis significantly in 10 of 16 cancers.

To further reveal the impact of *FYN* on the prognosis of kidney cancer, we also assessed the impact of CpG sites in the *FYN* promoter region on the prognosis of KIRP. Firstly, we also made differential analysis for CpG sites, but only one CpG site was significantly differential between tumour and normal samples, indicating that the differential levels of CpG sites in different cancers were specific and dynamic. As there was only one CpG site, we used the DNA methylation level of this CpG site as the risk score. We used the K-M survival curve and the logrank test to evaluate the prognosis of cancer patients after grouping by Maxstat, and found that the hypermethylated population could be distinguished from the hypomethylated population (*p* = 0.004), and the prognosis of hypermethylated population was better than that of hypomethylated population (Figure 8e). There were significant differences in methylation levels between hypermethylated and hypomethylated populations (*P* = 7.36 e-10, student’s T test) (Figure 8f). Through the analysis of *FYN* in KIRC and KIRP, we found that the dysregulation of *FYN* by DNA methylation could significantly affect the prognosis of cancer patients. And the differentially methylated CpG sites in KIRC and KIRP were different. Previous studies have demonstrated that although the regulatory CpG sites of different cancer promoter regions were specific and dynamic, their overall regulatory tendency for marker genes was stable [74]. We thought the traditional experiment should focus on all CpG sites in promoter region, but not only some CpG sites.

### The independent dataset validates the importance of DNA methylation

**Figure 9.**
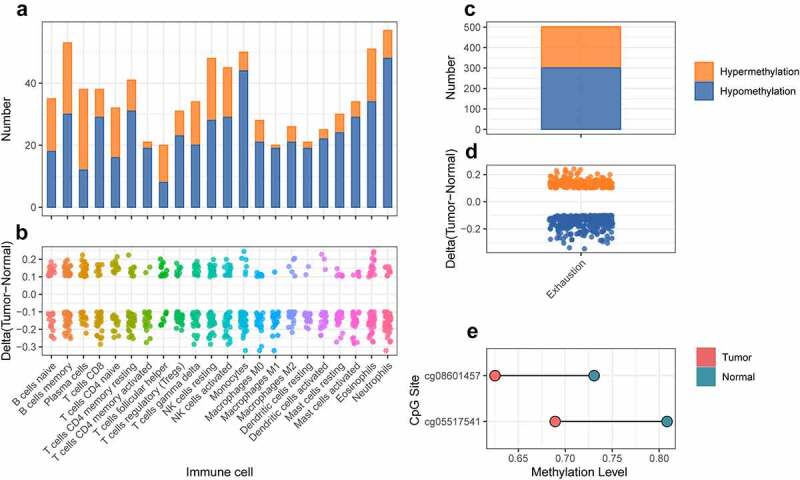
**The differential analysis of cross validation dataset for kidney cancer**. a. The number of differentially methylated sites for marker genes of immune cell in cross validation dataset. The orange and blue represented hypermethylation and hypomethylation, respectively. b. The delta value of differentially methylated sites for marker genes of immune cell in cross validation dataset. c. The number of differentially methylated sites for marker genes of T cell exhaustion in cross validation dataset. The orange and blue represented hypermethylation and hypomethylation respectively. d. The delta value of differentially methylated sites for marker genes of T cell exhaustion in cross validation dataset. e. The methylation level of differentially methylated sites for *FYN* between tumor and normal samples in cross validation dataset.

Then, we got another independent dataset (GSE113501) from GEO to demonstrate the importance and reliability of our analysis. Firstly, we made differential analysis to detect the methylation pattern. Next, the differentially methylated CpG sites of immune marker genes from CIBERSORT were extracted from the differential result. 19 out of 22 had more hypomethylated CpG sites in promoter regions than hypermethylated CpG sites (Figure 9a and b). The differentially methylated CpG sites of marker genes from T cell exhaustion were also extracted from the differential result. There were 201 hypermethylated and 301 hypomethylated CpG sites, respectively (Figure 9 Aand b). Finally, the CpG sites in *FYN* promoter region were also extracted from the differential result. Only two CpG sites (cg08601457 and cg05517541) were differential and they were both differentially hypomethylated.

These results further revealed the important role of DNA hypomethylation in tumour immune environment, and demonstrated the dynamic pattern of gene promoter region. Our study provided a foundation for the epigenetic study of tumour immune microenvironment.

## Discussion

In this work, we first characterized the global methylation level of cancer using the DNA methylation profiles from TCGA, and found that the global methylation level and the CpG island methylation level within the promoter were decreased, which proved that the abnormal methylation level results in the expression dysregulation for protein coding genes. Using regression analysis, a relationship between DNA methylation and protein coding genes was then calculated, and GSEA was used to perform a functional enrichment analysis. We found that they were enriched in biological processes, pathways, and hallmarks such as innate immune response, biological stimulus response, cell killing, viral response, humoral immunity, white cell-mediated immunity, interferon response, inflammatory response, and cytokine-to-cytokine interactions, which proved the important role of epigenetic regulation in immune response and cancer. Besides, a large majority of immune-related functions were covered in multiple cancers and were impacted by hypomethylation of the promoter region. These findings demonstrate the global functional stability of DNA hypomethylation interferences.

The association between DNA methylation level and immune infiltration in cancer patients was subsequently analysed. Hypomethylation was shown to contribute to more immune infiltration, especially for head and neck squamous cell carcinoma (HNSC: 16), kidney renal clear cell carcinoma (KIRC: 12), breast invasive carcinoma (BRCA: 8), kidney renal papillary cell carcinoma (KIRP: 7) and bladder urothelial carcinoma (BLCA: 5). Some of these cancers have responded to immune-checkpoint-blocking therapy in previous studies. To further estimate the role of DNA methylation in immunology, we used differentially methylated genes in kidney renal clear cell carcinoma to distinguish subtypes. The subtype with low methylation level showed a lower immune score and tumour mutation burden than subtypes with high methylation level. These results suggest that dysregulated methylation can influence the immune function and increased methylation may act as a biomarker to detect the response level to immunotherapy.

We also analysed DNA methylation level of exhaust-related marker genes previously mined in the literature and found 702 differentially methylated genes that were strongly associated with gene expression. By functional enrichment analysis of the marker genes, it was found that they were closely related to immunity and cancer. These results suggest that dysregulated genes are mediated by aberrant DNA methylation and involved in important biological functions for cancer. Next, the PPI network was constructed with the marker genes. Through the analysis of hub genes, we found that the hub genes were all abnormal marker genes, indicating that marker genes mediated by dysregulated DNA methylation were dominant in PPI network. We then used the hub gene *FYN* to perform survival analysis in KIRC and KIRP. Through the differential analysis between tumour and normal samples, 5 CpG sites and 1 CpG sites were differentially hypomethylated in KIRC and KIRP, respectively. These differential CpG sites were significantly associated with patients’ prognosis, and low-risk group had higher methylation level than high-risk group. These results suggest that dysregulated hypomethylation contributes to dysfunction of functional T cell, and disturbs the prognosis of patients.

Finally, we used a cross validation dataset of kidney cancer to demonstrate our results. We found hypomethylation also played important roles in tumour immune environment. Marker genes in immune cell and T cell exhaustion were mainly hypomethylated in tumour samples. And DNA methylation in promoter regions kept the aberrant hypomethylation level with a dynamic pattern.

In summary, dysregulated methylation represents an additional layer of immune system complexity. Analysing the mechanism of DNA methylation can help understand the immune microenvironment in cancer. Continued investigation of the immune-related methylation will help the development of better immunotherapies for human cancer and other diseases.

## Supplementary Material

Supplemental Material

## Data Availability

The data are available from TCGA database (https://portal.gdc.cancer.gov/).
