# Supplementary material for: DNA hypomethylation mediates immune response in pan-cancer
Source: Epigenetics. 2023 Mar 22;18(1):2192894. doi: 10.1080/15592294.2023.2192894 (PMC10038033; doi:10.1080/15592294.2023.2192894)
Supplement: Supplemental Material [file KEPI_A_2192894_SM2967.zip › Supplementary files/Supplementary legend.docx]

**Supplementary legend**

**Supplementary Figure 1. The sample information in 16 cancers.** A. The stage distribution of tumor malignant level in 16 cancers. B. The grade distribution of tumor malignant level in 16 cancers. The bar plot represented the percentage of each category. C. The age distribution in 16 cancers. The line plot represented the density of age.

**Supplementary Figure 2. The distribution of differentially methylated exhaustion-related marker genes.** A. The number of differentially hypermethylated genes in 16 cancers. B. The distribution of differentially hypermethylated genes in 16 cancers. C. The number of differentially hypomethylated genes in 16 cancers. D. The distribution of differentially hypomethylated genes in 16 cancers. The bar plot represented the number of differentially methylated genes. The y axis in heatmap represented marker genes, and red represented the gene was differential in each cancer.

**Supplementary Figure 3. The survival result of hub genes in 16 cancers.** The yellow represented the gene was associated with prognosis significantly.

**Supplementary Table 1. The list of exhaustion-related genes.**
