# Supplementary figures and images for: DNA hypomethylation mediates immune response in pan-cancer
Source: Epigenetics. 2023 Mar 22;18(1):2192894. doi: 10.1080/15592294.2023.2192894 (PMC10038033; doi:10.1080/15592294.2023.2192894)

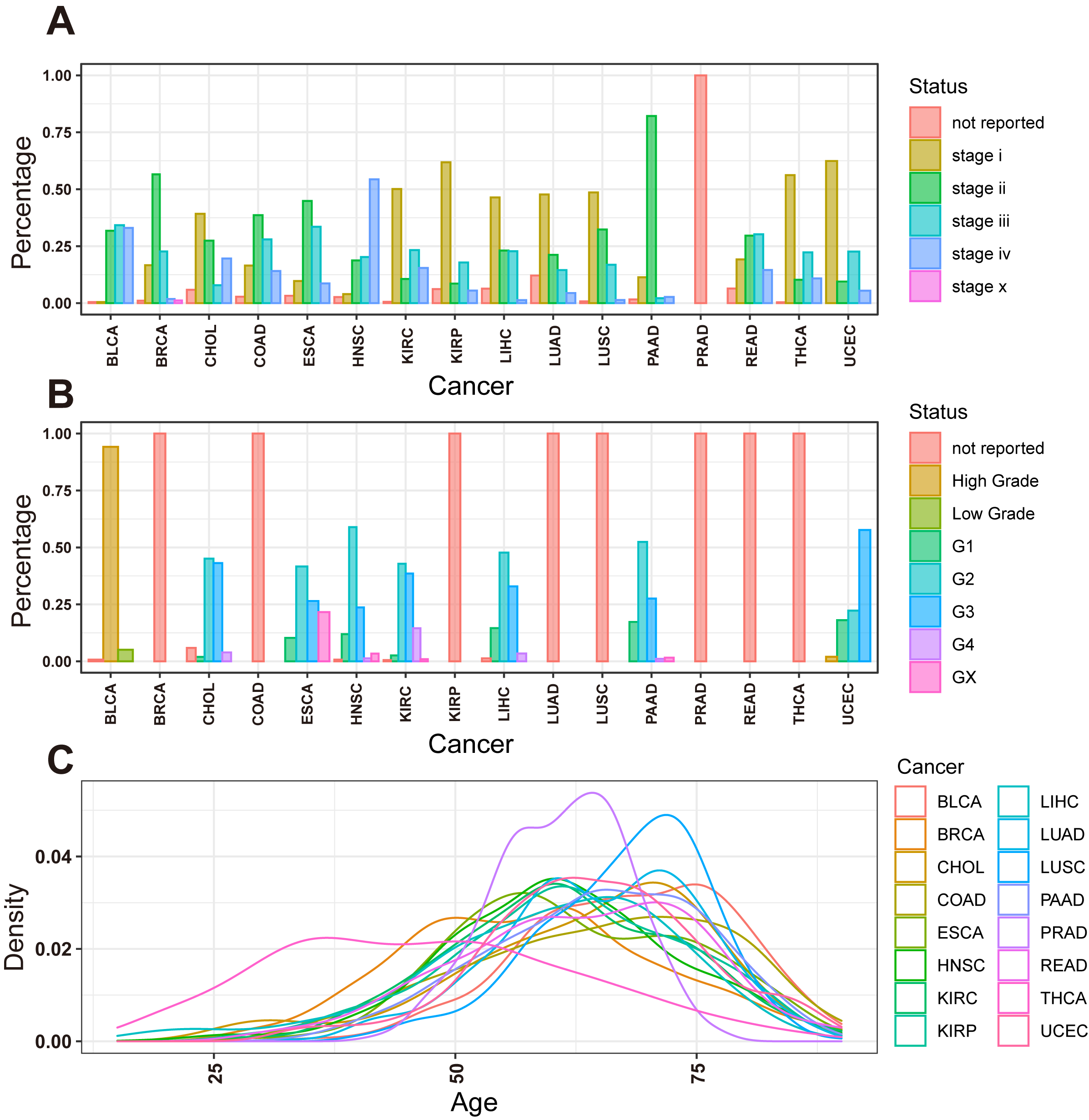

Supplement: Supplemental Material [file KEPI_A_2192894_SM2967.zip › Supplementary files/Supplementary figure 1.tif]

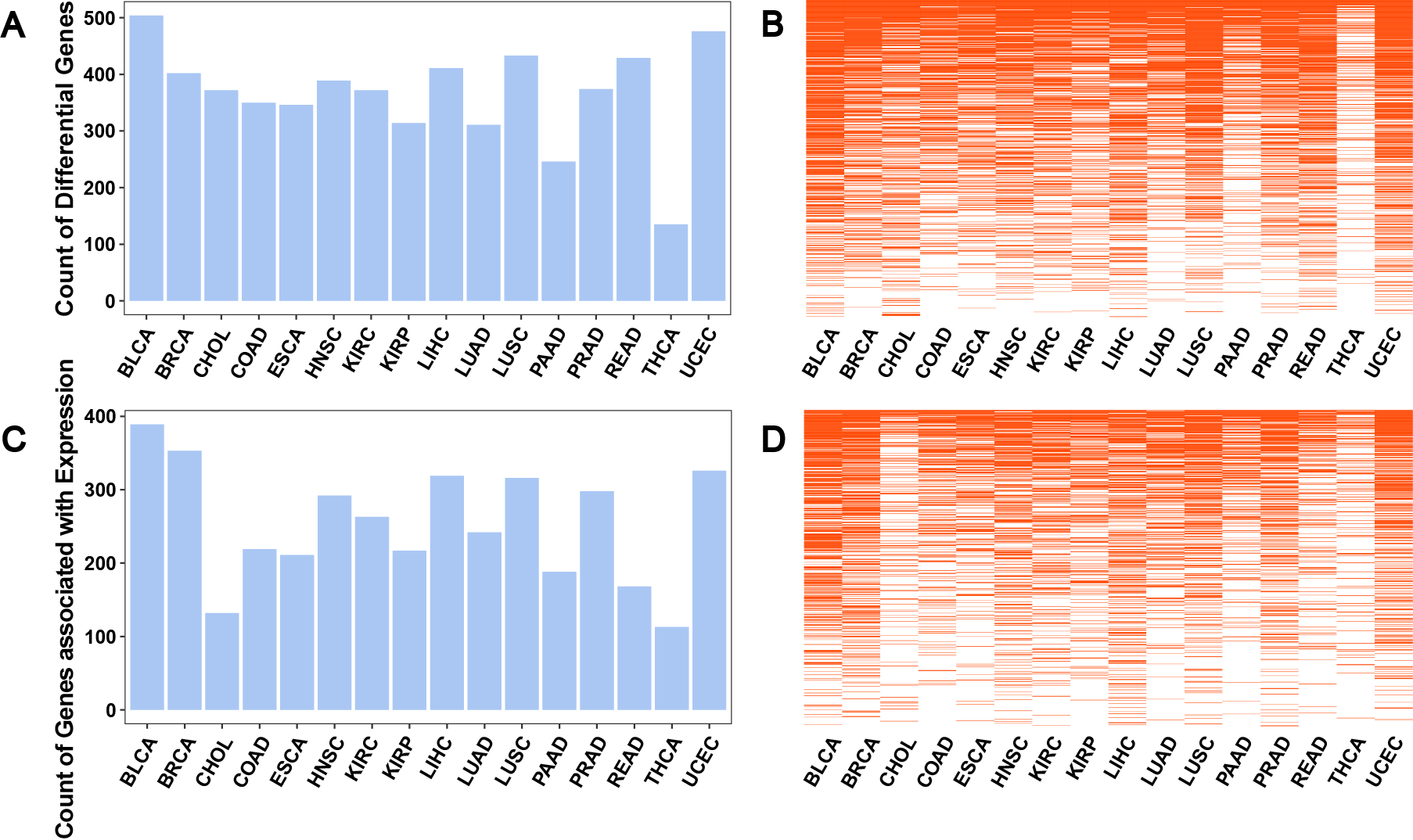

Supplement: Supplemental Material [file KEPI_A_2192894_SM2967.zip › Supplementary files/Supplementary figure 2.tif]

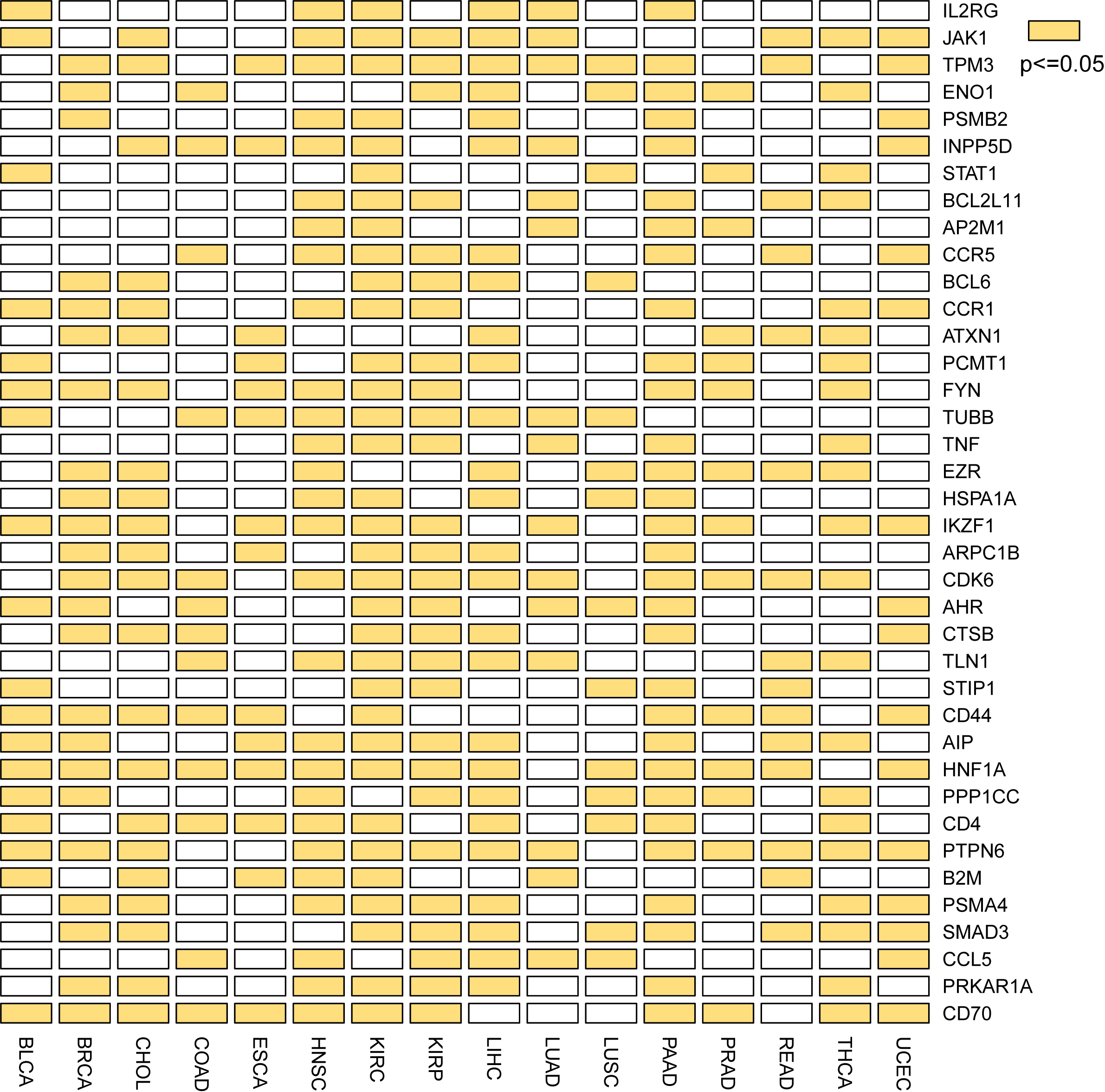

Supplement: Supplemental Material [file KEPI_A_2192894_SM2967.zip › Supplementary files/Supplementary figure 3.tif]
